# Non-syndromic *OTX2*-associated pattern dystrophy: a 10-year multimodal imaging study

**DOI:** 10.1007/s10633-024-09983-w

**Published:** 2024-07-18

**Authors:** Prathiba Ramakrishnan, Matthew K. Kenworthy, Jonathan A. Alexis, Jennifer A. Thompson, Tina M. Lamey, Fred K. Chen

**Affiliations:** 1 Ocular Tissue Engineering Laboratory, Lions Eye Institute, Nedlands, WA Australia; 2 Australian Inherited Retinal Disease Registry and DNA Bank, Department of Medical Technology and Physics, Sir Charles Gairdner Hospital, Nedlands, WA Australia; 3 Centre for Ophthalmology and Visual Science, The University of Western Australia, Perth, WA Australia; 4 Centre for Eye Research Australia, Royal Victorian Eye and Ear Hospital, Melbourne, VIC Australia; 5 Department of Surgery, University of Melbourne, Melbourne, VIC Australia; 6 Lions Eye Institute, Perth, WA Australia

**Keywords:** Macular dystrophy, Multimodal imaging, Microperimetry, Avascular retina, Megalopapilla

## Abstract

**Purpose:**

To report novel multimodal imaging features and long-term follow-up of Orthodenticle Homeobox 2 (*OTX2*)-associated pattern Gdystrophy.

**Methods:**

A 14-year-old boy referred with glaucoma suspect and macular pigmentation underwent fundus autofluorescence imaging, optical coherence tomography, fluorescein and indocyanine green angiography, visual field test, microperimetry and electrophysiology over a ten-year period. Next-generation sequencing panel identified a de novo heterozygous likely pathogenic *OTX2* variant, c.259G>A, [p.(Glu87Lys)].

**Results:**

Visual acuity was 20/40 OD and 20/30 OS. Examination showed bilateral enlarged optic nerve heads and increased disc cupping, multiple cilioretinal arteries, a pigmentary maculopathy with stellate-shaped region of hypoautofluorescence, shallow serous macular detachment, subretinal deposits and temporal avascular retina. Angiography showed no source of leakage and absence of retinal neovascularisation despite extensive peripheral non perfusion. Electrophysiological assessments demonstrated mild progressive rod and cone pathway abnormalities, reduced light-adapted b:a ratio, and reduced Arden ratio on electro-oculogram. Ten-year follow-up confirmed a stable disease course despite persistent submacular fluid. There was no associated pituitary structural abnormality or dysfunction.

**Conclusions:**

This case study contributes to further understanding of *OTX2*-associated pattern dystrophy, highlighting its stability over 10 years. Further investigation into inter-individual and intrafamilial variability is warranted.

**Supplementary Information:**

The online version contains supplementary material available at 10.1007/s10633-024-09983-w.

## Introduction

Orthodenticle Homeobox 2 (*OTX2*) is a master gene on chromosome 14q22.3 coding for a critical transcription factor involved in the embryonic development of the forebrain and the eye including photoreceptors, bipolar cells, and retinal pigment epithelial (RPE) precursor cells [1, 2]. Detailed multimodal imaging features of *OTX2*-associated pattern dystrophy and its long-term outcomes are lacking due to its rarity and short observation time in previous studies [3–5]. Herein, we report novel phenotypic features of *OTX2*-associated retinopathy and the 10-year functional and structural outcomes in a 25-year-old male who has undergone multimodal imaging since the age of 14.

## Clinical presentation

**Fig. 1:**
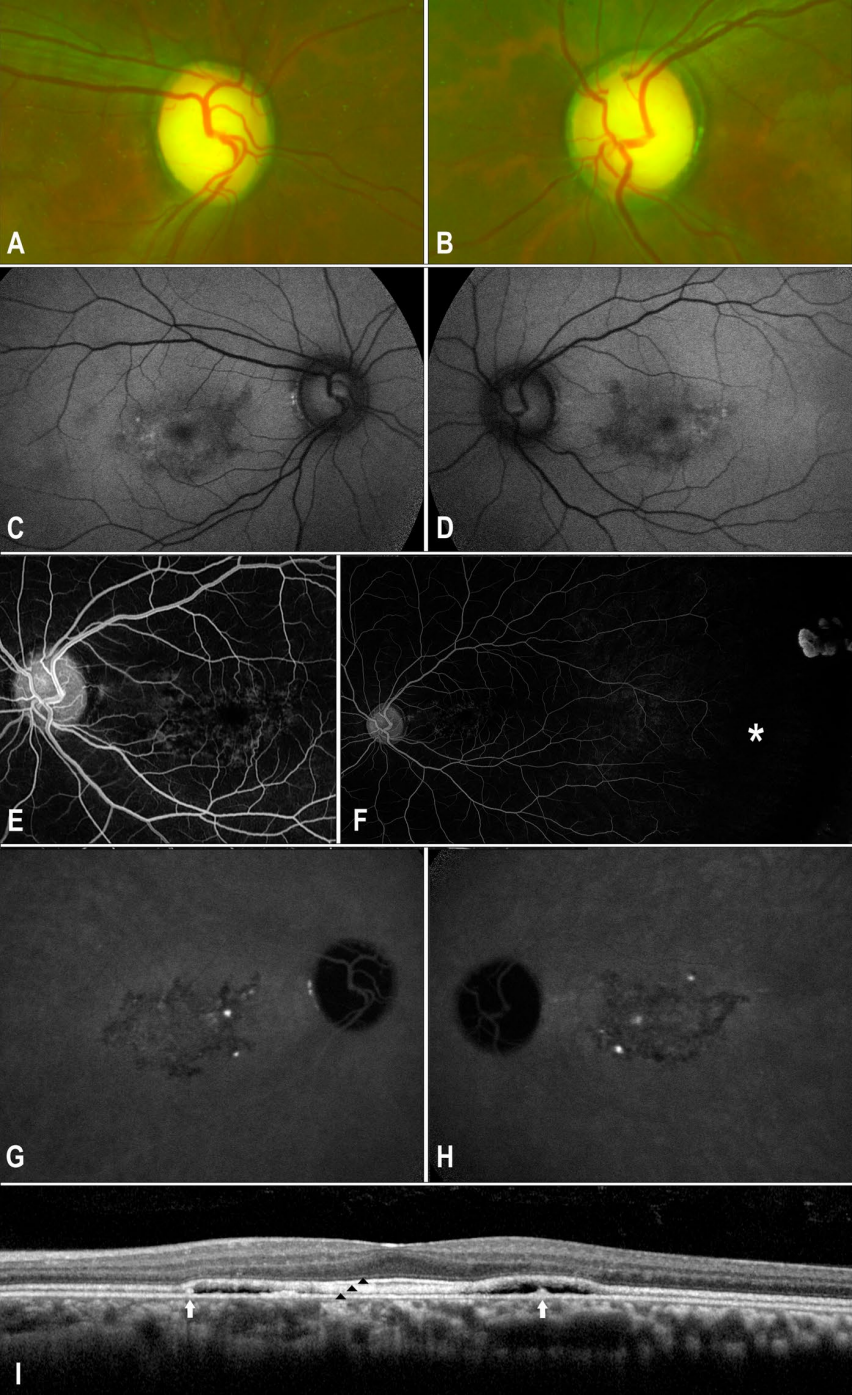
Pseudocolour optic disc photos reveal megalopapillae with physiological disc cupping in both eyes at the initial presentation (**A, B**). Short-wave fundus autofluorescence displays a central irregular-shaped hypoautofluorescent region with mild perifoveal hyperautofluorescence in both eyes (**C, D**). Fundus fluorescein angiography (FFA) of the left eye (mid-phase) shows macular stippling (**E**). Ultrawide-field FFA demonstrates peripheral avascularity (marked by the white star) and retinochoroidal atrophy without neovascularization (**F**). Indocyanine Green dye test exhibits multifocal hyperfluorescent areas without leakage in the late phase (**G**,** H**). Heidelberg spectral-domain optical coherence tomography (OCT) after 10 years of follow-up demonstrates shallow persistent foveal detachment with a thickened ellipsoid zone and subretinal deposits (shown by the white arrow, (**I**). The ellipsoid zone remains fused with the interdigitation zone, except at the fovea where the latter became more prominent (indicated by three black arrowheads)

A 14-year-old boy of Indian origin presented with incidental bilateral macular pigmentary changes whilst being evaluated for suspected glaucoma. He had delayed speech onset at 2 years of age and myopia was noted at age 3. His vision was 20/40 OD and 20/30 OS. Intraocular pressures were 13 mmHg OD and 12 mmHg OS with open angles on gonioscopy. Fundoscopy revealed macular pattern dystrophy, megalopapilla with physiological disc cupping, multiple cilioretinal arteries, and temporal peripheral retinal avascularity bilaterally (Table 1, Fig. 1A and B).

**Table 1:** A summary of baseline clinical findings

Right eye	Ocular findings	Left eye
20/40	Visual acuity (Snellen)	20/30
−4.75/−2.25 × 30°	Refractive error	−4.75/−1.50 × 152°
590	Central corneal thickness (micrometres)	K588
26.46	Axial length (mm)	26.31
11.8	White-to-white (mm)	11.8
12	Intraocular pressure (mmHg)	13
Peripheral corneal stromal scars, clear lens	Anterior segment	Peripheral corneal stromal scars, clear lens
Normal vitreous Vertical disc diameter 2.2 mm Vertical cup disc ratio 0.70 Macular yellow spots Avascular peripheral retina	Posterior segment	Normal vitreous Vertical disc diameter 2.3 mm Vertical cup disc ratio 0.65–0.70 Macular yellow spots Avascular peripheral retina
Central hypoautofluorescence surrounded by hyper autofluorescence	Fundus autofluorescence (BAF 30°)	Central hypoautofluorescence surrounded by hyper autofluorescence
Macular serous detachment with subretinal hyperreflective deposits and fused ellipsoid layer	Optical coherence tomography	Macular serous detachment with subretinal hyperreflective deposits and fused ellipsoid layer
Normal	Visual field and microperimetry	Normal

*BAF* blue autofluorescence

Short-wave fundus autofluorescence (AF) showed a central stellate-shaped region of hypoautofluorescence with mild perifoveal hyperautofluorescence in both eyes (Fig. 1C and D). Spectral-domain optical coherence tomography (OCT, Heidelberg Spectralis, Heidelberg Engineering, Heidelberg, Germany) showed shallow neurosensory detachment with ellipsoid and interdigitation zone fusion resulting in apparent thickening of band 2 in the perifoveal area. Interdigitation zone thickening was notable in the foveal region. An ultrawide-field fluorescein angiography (Optos, Dunfermline, Scotland) revealed stippled foveal hyperfluorescence and multiple cilioretinal arteries originating from the nasal and superior discs, together with far-temporal avascular retina (Fig. 1E and F). Indocyanine green angiography showed abnormal macular choroidal vasculature with two intensely hyperfluorescent lesions within the fovea that corresponded to focal thickening of the RPE layer (Fig. 1G and H). There was no angiographic leakage.

**Fig. 2:**
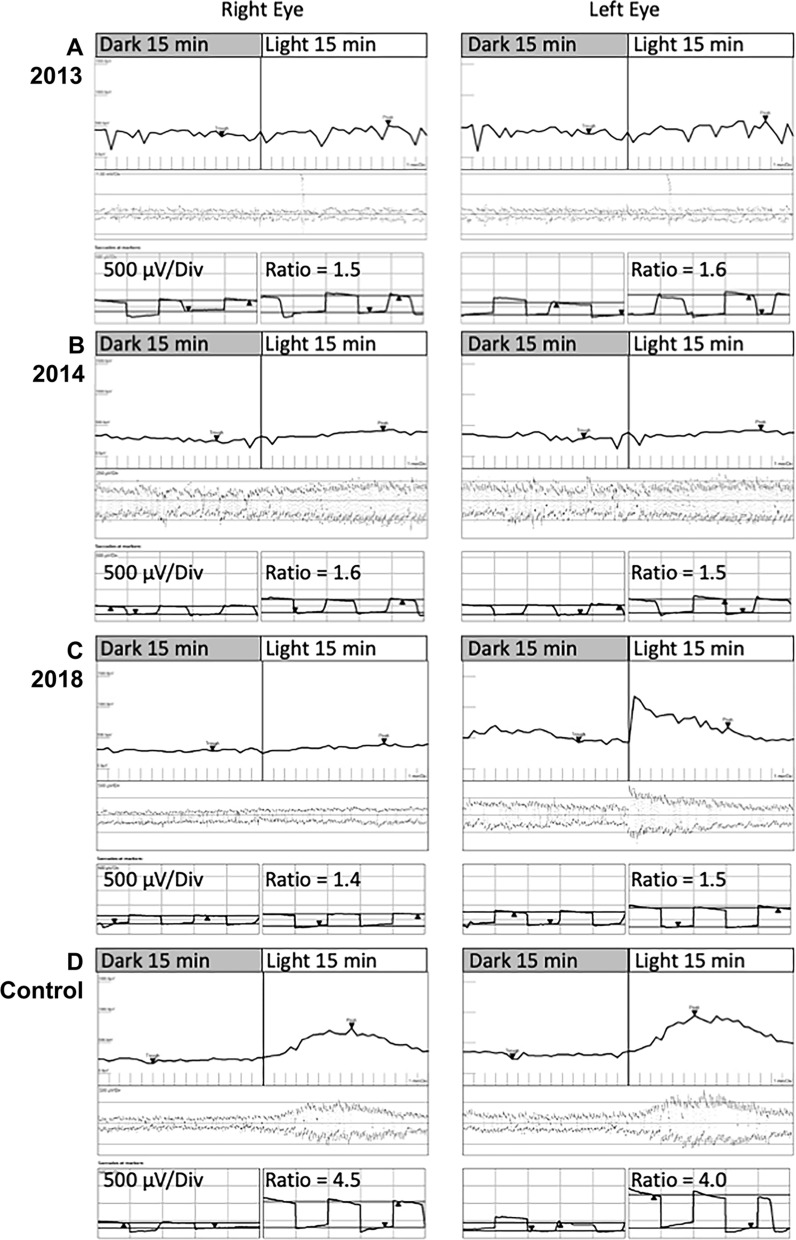
Electro-oculogram of patient in 2013 (**A**), 2014 (**B**) and 2018 (**C**) show the average amplitude at each minute of recording and the measurements of the light peak to dark trough ratio for both eyes compared to Electro-oculogram of a healthy individual (**D**)

**Fig. 3:**
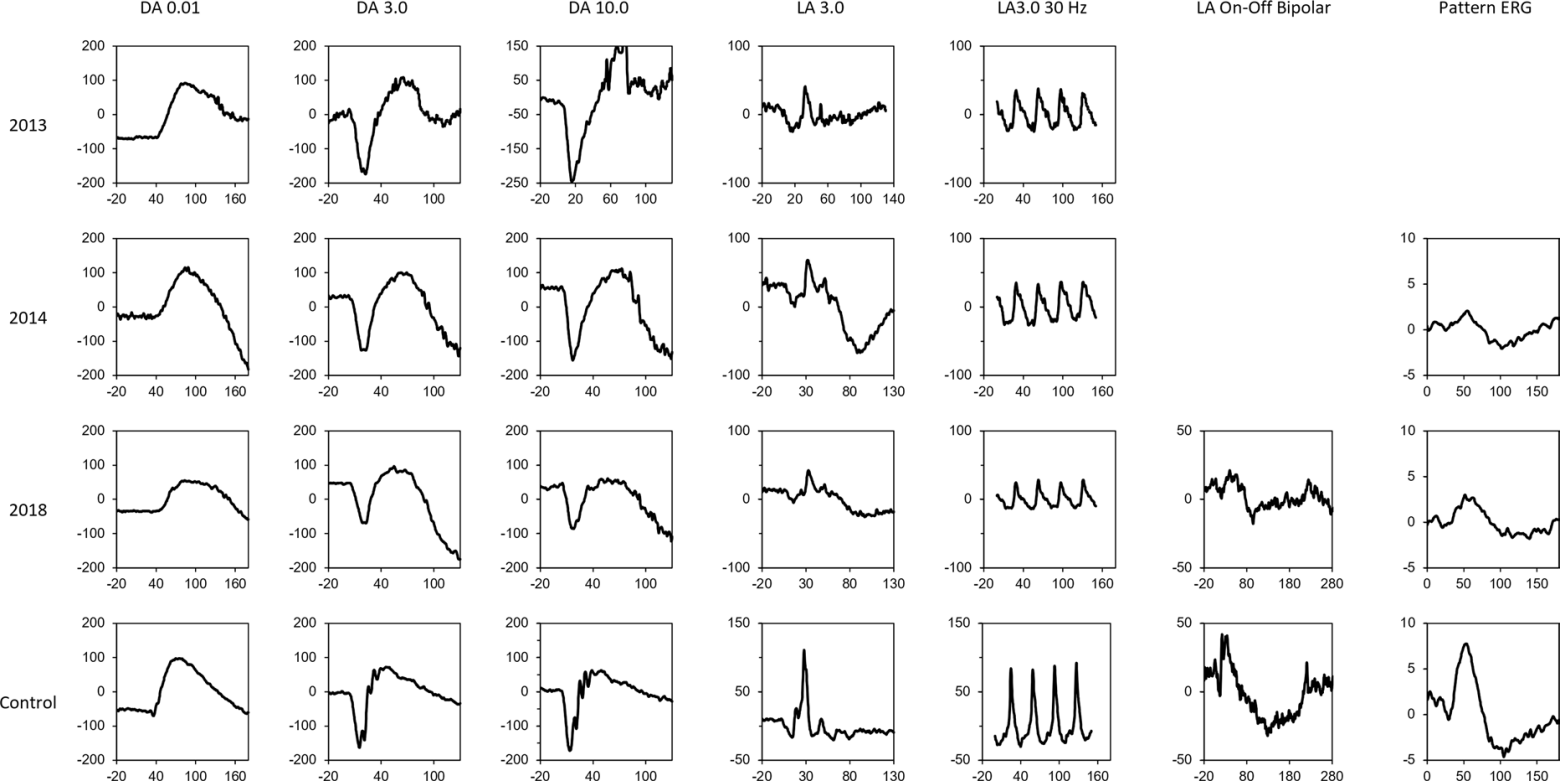
Electroretinogram of patient in 2013, 2014, 2018 against an age-similar control subject. DA 0.01: dark-adapted 0.01 cd s m^−2^; DA 3.0: dark-adapted 3.0 cd s m^−2^; DA 10.0: dark-adapted 10.0 cd s m^−2^; LA 3.0: light-adapted 3.0 cd s m^−2^ at 30 cd.m^−2^ background; LA 30 Hz: light-adapted 3.0 cd s m^−2^ flicker at 30 cd m^−2^ background; LA On–Off Bipolar: light adapted on–off response

Humphrey visual field examinations were within normal limits OU. Baseline microperimetry (Macular Integrity Assessment, MAIA, CenterVue, Padova, Italy) showed focal shallow scotoma across the macular region (Supp. Fig. S1A and S1B, online source 1). Visual electrophysiology (RETIport 3.2, Roland Consult, Brandenburg, Germany) was performed incorporating the 2008 standards as set out by the International Society for Clinical Electrophysiology of Vision (ISCEV) [6]. Electro-oculography performed in accordance to the ISCEV standard [7] revealed an Arden ratio of 1.5 OD and 1.6 OS (normal ≥ 1.7, Fig. 2) in accordance with the ISCEV Standard for Clinical Electro-oculography Full-field ERG showed dark-adapted (DA) responses were within the normal range at baseline (Supp. Table S1, online source 2). Light-adapted (LA) 30 Hz flicker was reduced and delayed. Reduced b-wave amplitude and reduced b:a ratio (2.8 OD, 2.1 OS, normal ≥ 3.0) in the LA3.0 standard flash indicated abnormalities in the inner retinal cone pathway (Fig. 3).

Magnetic resonance imaging and endocrine work up (serum electrolytes, thyroid stimulating hormone, prolactin, insulin-like growth factor 1, cortisol, follicular stimulating hormone and luteinizing hormone) excluded pituitary structural abnormality and dysfunction.

## Molecular diagnosis

Genetic analysis was performed through the Australian Inherited Retinal Diseases Registry and DNA Bank. Genomic DNA was extracted from peripheral blood as described previously [8]. Next-generation sequencing was performed (Retinal Dystrophy Smart panel, version 8; Casey Eye Institute Molecular Diagnostic laboratory (CEI), Portland, Oregon, USA) targeting all exons and flanking intronic sequences of 244 retinal dystrophy genes [9]. Sanger sequencing was used to confirm candidate variants in the proband and for phase testing in parental DNA (CEI). Genetic analysis of the proband identified a heterozygous *OTX2* variant, NM_001270525.1:c.259G>A, [p.(Glu87Lys)], previously reported as a dominant variant published as NM_001270523.1:c.235G>A, [p.(Glu79Lys)] by Vincent et al. [3] phase testing of parental DNA showed neither parent carried the variant. We have assessed this variant as likely pathogenic (PM6_SUPP, PS4_MOD, PM2_SUPP, PP3_STR) according to ACMG standards and guidelines and associated literature (Supp. Table S2, online source 3) [10–13]. We have not applied the PP5 rule, “reputable source recently reports variant as pathogenic, but the evidence is not available to the laboratory to perform an independent evaluation”, as recommended by Biesecker and Harrison [14].

## Follow-up Imaging

Ten years after presentation, the patient reported declining vision in the right eye. At 25, his visual acuities were 20/50 OD and 20/30 OS and IOP was 19 mmHg OD and 20 mmHg OS without topical glaucoma treatment. There was no clinical or OCT progression. Subretinal fluid (SRF) fluctuated over the 10 years with central subfield thickness (CST) ranging from 288 to 368 µm, unresponsive to oral acetazolamide. Some subretinal deposits resolved, whilst others remained. The ellipsoid zone remained fused with the interdigitation zone except at the fovea where the latter remained prominent (Fig. 1I). The most recent MAIA showed a mean sensitivity of 26.6 dB OD and 26.4 dB OS (Supp. Fig. S1C and S1D). At age 20, 5 years after baseline, follow-up electrophysiology showed a further reduction in the Arden ratio (1.4 OD, 1.5 OS, normal ≥ 1.7) whilst full-field ERG showed additional rod pathway abnormality at the photoreceptor level and cone pathway dysfunction at both photoreceptor and post-photoreceptor levels (reduced LA3 b:a ratios of 2.52 OD and 2.94 OS). On–off response showed a reduced b-wave response (24 µV OD, 20 µV OS, normal 28–63 µV) indicating an on-bipolar cell abnormality (Figs. 2 and 3, Supp. Table S1).

## Discussion

*OTX2*-associated eye disease is rare and is known for its association with pituitary abnormalities and diverse ocular phenotypes [2–4, 15]. Although 52 variants have been listed in the Leiden Open Variation Database (LOVD), only 30 are considered pathogenic or likely pathogenic. *OTX2* disease-causing variants are more commonly known for anophthalmia, microphthalmia, anterior segment dysgenesis, optic nerve dysplasia/hypoplasia and combined pituitary hormone deficiency, with an overall estimated frequency of ocular anomalies as high as 86% [3, 15–19]. The *OTX2* variant, c.259G > A, was previously detected by exome sequencing (234 genes) of DNA from 298 probands diagnosed with early-onset high myopia [20]. However, there was no phenotypic data except for the presence of high myopia defined by at least −6.0D or axial length of > 26 mm before the age of seven. Vincent et al. found the same *OXT2* variant heterozygously in two families with autosomal dominant pattern dystrophy [3]. They noted optic nerve head dysplasia and microcornea with variable photoreceptor abnormalities on ERG. The detection of anomalous optic nerve head prompted ophthalmic assessment in our case. Interestingly, the presence of megalopapilla, alongside physiologic disc cupping and incidentally elevated IOP, has not been documented previously as presenting features of *OTX2*-associated eye disease. Up to 51% of individuals with *OTX-2*-associated eye disease had brain and pituitary abnormalities [21]. However, despite optic nerve head irregularities, our patient showed no associated brain or pituitary changes. This observation is consistent with the previous report by Vincent et al. [3]. The variable brain versus ocular phenotype may be related to unique brain isoform function which is unaffected by the missense variant specific for a pattern dystrophy.

Previous reports of *OTX2*-related macular pattern dystrophy described shallow retinal elevation on OCT but lacked detailed multimodal imaging or long-term follow-up [3, 4, 19]. We noted an irregular geographic region of poorly defined macular hypoautofluorescence with pericentral hyperautofluorescence. These changes were symmetrical with normal peripheral retinal AF signals in both eyes. In our case, we noted shallow SRF accompanied by subretinal hyperreflective deposits. Despite oral acetazolamide treatment, the SRF exhibited mild fluctuations and remained unresponsive. While certain subretinal deposits resolved, others remained stable. Additionally, fusion of the ellipsoid and interdigitation zones resulted in a distinctive thickened second band on OCT, characteristic of *PRPH2*-associated retinopathy [22]. An atypical feature in this case is the separation of the interdigitation zone from band 2 and thickening of band 3 in the fovea. These separations could be attributed to the role of OTX2 in preserving the contact between photoreceptors and the RPE over time, a phenomenon that has also been demonstrated in a knockout mouse model [23]. Indocyanine green angiography revealed multifocal staining during the mid-phase co-localized with subretinal deposits. However, there was no obvious leak during transit to reveal the source of SRF. One may hypothesize that SRF is a sign of RPE pump insufficiency resulting from an *OTX2*-related developmental anomaly. Further unique findings included peripheral avascularity resembling familial exudative vitreoretinopathy and retinopathy of prematurity without vessel straightening, macular traction, retinal neovascularization, or a history of prematurity. Incomplete vascularisation of the peripheral retina has been reported in the microphthalmia eyes of a 25-month-old female harbouring the *OTX2* variant, c.135dupA, [p.(Thr46AsnfsTer84)]. There was no evidence of retinal neovascularisation at 10-years of follow-up even without laser photocoagulation to areas of avascularity.

Detailed electrophysiological assessment demonstrated a progressive RPE dysfunction over a 5-year interval as shown by the declining Arden ratio (Fig. 2). This trend aligns with cross sectional findings of marginal EOG parameter in young children and significantly lower values in young adults [3]. The progressive reduction in EOG values may be linked to the role of OTX2 in RPE function as evidenced by a knockout mice model [3, 23]. Studies have previously found rod and cone photoreceptor pathway abnormalities including On- and Off-bipolar pathway alterations and S-cone pathway preservation [3, 5]. We observed progressive rod and cone photoreceptor abnormalities with additional post-photoreceptor dysfunction in the cone pathway (Supp. Table S1). Profound inner retinal dysfunction has been reported previously in a 7-year-old male with infantile onset retinal dystrophy resulting from the heterozygous *OTX2* variant, c.413C>G, [p.(Ser138Ter)] [5]. These findings are consistent with the known role of OTX2 in photoreceptor and bipolar cell development and terminal differentiation [24, 25]. The declining LA3.0 a-wave amplitude may be related photoreceptor degeneration due to the chronic shallow submacular fluid secondary to RPE dysfunction. However, there was no multimodal imaging changes to indicate rod photoreceptor loss as suggested by the declining DA3.0 a-wave amplitude.

In conclusion, we present a case of *OTX2*-associated macular pattern dystrophy with megalopapilla, retinal vascular anomalies and relatively stable disease course. Despite persistent shallow subretinal fluid, there was no RPE atrophy or macular sensitivity decline. Progressive generalised rod and cone system, and RPE dysfunction was evident despite SRF being limited to the macula and preserved peripheral fundus autofluorescence signal. Further long-term follow-up studies are warranted to determine the mechanism for functional decline in the absence of structural change.

## Supplementary Information

Below is the link to the electronic supplementary material.

Supplementary file1 (DOCX 463 KB)

Supplementary file2 (DOCX 18 KB)

Supplementary file3 (DOCX 22 KB)

## Declarations

### Statement on the welfare of animals

No animal was used in this study.
