# Supplementary material for: Non-syndromic OTX2-associated pattern dystrophy: a 10-year multimodal imaging study
Source: Doc Ophthalmol. 2024 Jul 18;149(2):115–23. doi: 10.1007/s10633-024-09983-w (PMC11442598; doi:10.1007/s10633-024-09983-w)
Supplement: Supplementary file 1 — Supplementary file1 (DOCX 463 KB) [file 10633_2024_9983_MOESM1_ESM.docx]

**Supplementary Figure S1**


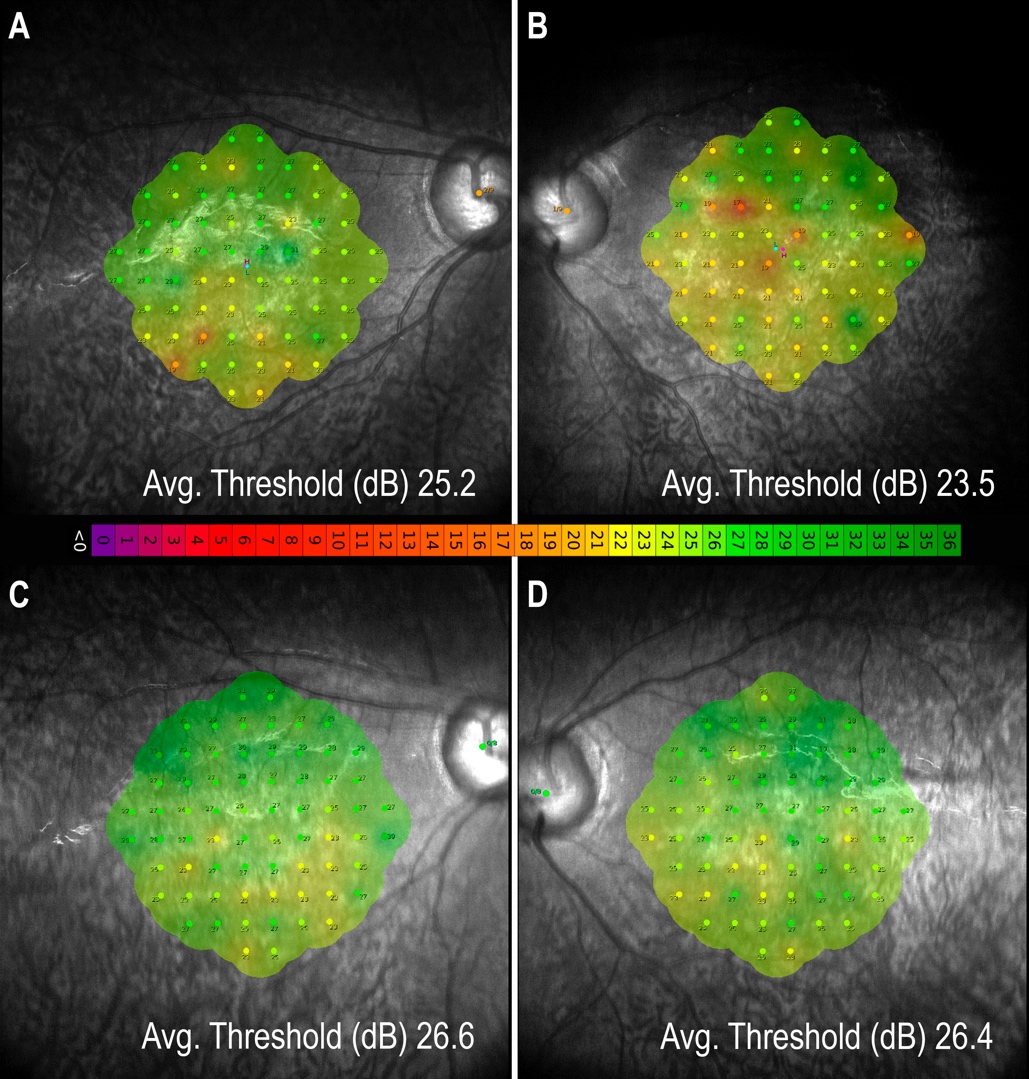


**Figure legend.**

Baseline MAIA microperimetry of the right and left eyes demonstrates a slightly reduced average threshold in the left eye (**A, B**). Follow-up MAIA after a 10-year interval shows preserved macular sensitivity despite persistent subretinal fluid throughout the follow up period (**C, D**).
