# Supplementary material for: Non-syndromic OTX2-associated pattern dystrophy: a 10-year multimodal imaging study
Source: Doc Ophthalmol. 2024 Jul 18;149(2):115–23. doi: 10.1007/s10633-024-09983-w (PMC11442598; doi:10.1007/s10633-024-09983-w)
Supplement: Supplementary file 2 — Supplementary file2 (DOCX 18 KB) [file 10633_2024_9983_MOESM2_ESM.docx]

**Supplementary Table S1. Visual electrophysiology component measurements 2013-2018**

| **ERG components** | **Right Eye** | | | **Normal Range** | **Left Eye** | | | **Change over time** |
| --- | --- | --- | --- | --- | --- | --- | --- | --- |
|  | **2013** | **2014** | **2018** |  | **2013** | **2014** | **2018** |  |
| **DA0.01** | | | | | | | | |
| b-wave  amplitude (µV) | 234 | 146 | 92* | 103-300 | 154 | 142 | 87* | Reduced to outside NR |
| b-wave  peak time (ms) | 97 | 100 | 85 | 82-103 | 83 | 89 | 90 | Stable |
| **DA3.0** | | | | | | | | |
| a-wave  amplitude (µV) | 141 | 149 | 115* | 125-238 | 170 | 155 | 110* | Reduced to outside NR |
| a-wave  peak time (ms) | 23 | 22.3 | 22.6 | 21-23 | 22 | 22.6 | 22.6 | Stable |
| b-wave  amplitude (µV) | 210 | 218 | 159* | 213-463 | 240 | 226 | 161* | Reduced to outside NR |
| b-wave  peak time (ms) | 50 | 62 | 54 | 48-55 | 49 | 62 | 53 | Stable |
| b:a ratio | 1.49 | 1.46 | 1.38 | ≥ 1.2 | 1.41 | 1.46 | 1.46 | Stable |
| **DA10.0** | | | | | | | | |
| a-wave  amplitude (µV) | 218 | 200 | 139 | 134-289 | 229 | 201 | 134* | Reduced to outside NR |
| a-wave  peak time (ms) | 16 | 17.3 | 16.7 | 15-18 | 17 | 17.0 | 16.7 | Stable |
| b-wave  amplitude (µV) | 295 | 252 | 196 | NA | 284 | 261 | 185 | Reduced but within NR |
| b:a ratio | 1.35 | 1.26 | 1.41 | ≥ 1.2 | 1.24 | 1.30 | 1.38 | Stable |
| **LA30Hz** | | | | | | | | |
| N1-P1  amplitude (µV) | 49.9* | 50.4* | 36.4* | 51-145 | 67.4 | 59.8 | 47.3* | Reduced to outside NR |
| P1  Peak time (ms) | 28.2 | 28.8* | 30.6* | 24.9-28.3 | 28.5* | 28.5* | 28.8* | Increased to outside NR |
| **LA3.0** | | | | | | | | |
| a-wave  amplitude (µV) | 17.6 | 22.3 | 16.0* | 17-39 | 32.2 | 22.8 | 15.3 | Reduced to outside NR |
| a-wave  peak time (ms) | 17.0* | 17.6* | 17.0* | 14-15 | 17.0* | 17.6* | 16.4* | Outside NR |
| b-wave  amplitude (µV) | 49.5* | 57.7* | 40.3* | 78-172 | 65.9* | 60.6* | 45.0* | Outside NR |
| b-wave  peak time (ms) | 32.0* | 32.3* | 33.8* | 28-31 | 32.0* | 32.0* | 32.9* | Outside NR |
| b:a ratio | 2.81* | 2.59* | 2.52* | ≥ 3.0 | 2.05* | 2.66* | 2.94* | Outside NR |
| **Arden Ratio** |  |  |  |  |  |  |  |  |
| **Light peak: Dark trough** | 1.5 | 1.6 | 1.4 | ≥ 1.7 | 1.6 | 1.5 | 1.5 | Outside NR |

NR, Normative range; NA, not available; DA, dark-adapted; LA, light-adapted
