# Supplementary material for: Non-syndromic OTX2-associated pattern dystrophy: a 10-year multimodal imaging study
Source: Doc Ophthalmol. 2024 Jul 18;149(2):115–23. doi: 10.1007/s10633-024-09983-w (PMC11442598; doi:10.1007/s10633-024-09983-w)
Supplement: Supplementary file 3 — Supplementary file3 (DOCX 22 KB) [file 10633_2024_9983_MOESM3_ESM.docx]

**Supplementary Table S2. ACMG rules applied for *OTX2* variant, NM_001270525.1:c.259G>A, with modified evidence level and justification**

| ACMG Rule | Description | Applied Rule Evidence | Justification |
| --- | --- | --- | --- |
| PM6 | Assumed *de novo*, but without confirmation of paternity and maternity. (phenotype matches gene with reasonable specificity) | PM6_SUPP | Variant detected in proband DNA and not parental DNA.  For supporting evidence: phenotype consistent with gene but not highly specific, as per SVI working group recommendation for *De Novo* Criteria (Version 1.1). |
| PS4 | The prevalence of the variant in affected individuals is significantly increased compared with the prevalence in controls (Odds ratio/relative risk >5 and CI <1; case-control studies) | PS4_MOD | Variant absent from gnomAD and previously identified as causative for the same phenotype (pattern dystrophy) in two unrelated pedigrees. [3] Case-control analysis not possible (rare variant).  For moderate evidence: present in ≥2 individuals for autosomal dominant disorder with high penetrance, as per ACGS Best Practice Guidelines for Variant Classification. [12,13] Only unrelated probands with a phenotype consistent with the disease (but not highly specific) are counted. |
| PM2 | Absent from controls, or at extremely low frequency if recessive, in Genome Aggregation Database, gnomAD | PM2_SUPP | Variant absent from gnomAD  Supporting evidence level only: as per recommended weight adjustment for this rule by SVI Working Group (SVI recommendation for Absence/Rarity Criterion, PM2, Version 1.0). |
| PP3 | Computational evidence supports a deleterious effect on the gene or gene product (conservation, evolutionary, splicing impact, etc.) | PP3_STR | The score generated by metapredictor, REVEL, for this missense variant is 0.951.  For strong evidence: REVEL score ≥0.932, as per ClinGen recommendations for PP3/BP4 criteria.[11] |

Underlined letters ‘P’, ‘M’, or ‘S’ and abbreviations ‘SUPP’ ‘MOD’ or ‘STR’ correspond to supporting, moderate, or strong levels of evidence, respectively as per ACMG rules and their description.[10]

**Table References**

[3] Vincent A, Forster N, Maynes JT, et al. OTX2 mutations cause autosomal dominant pattern dystrophy of the retinal pigment epithelium. *J Med Genet*. 2014;51(12):797-805.

[10] Richards S, Aziz N, Bale S, et al. Standards and guidelines for the interpretation of sequence variants: a joint consensus recommendation of the American College of Medical Genetics and Genomics and the Association for Molecular Pathology. Genetics in Medicine 2015;**17**(5):405-24 doi: 10.1038/gim.2015.30

[11] Pejaver V, Byrne AB, Feng BJ, et al. Calibration of computational tools for missense variant pathogenicity classification and ClinGen recommendations for PP3/BP4 criteria. Am J Hum Genet 2022;**109**(12):2163-77 doi: 10.1016/j.ajhg.2022.10.013

[12] Durkie M, Cassidy EJ, Berry I, Owens M, Turnbull C, Scott RH, Taylor RW, Deans ZC, Ellard S, Baple EL, McMullen DJ. ACGS Best Practice Guidelines for Variant Classification in Rare Disease 2024. [Best Practice Guidelines - The Association for Clinical Genomic Science (acgs.uk.com)](https://www.acgs.uk.com/quality/best-practice-guidelines/#VariantGuidelines)

[13] Zhang J, Yao Y, He H, Shen J. Clinical Interpretation of Sequence Variants. Curr Protoc Hum Genet. 2020 Jun;106(1):e98. doi: 10.1002/cphg.98. PMID: 32176464; PMCID: PMC7431429.
